# Rethinking the risk for depression using the RDoC: A psychophysiological perspective

**DOI:** 10.3389/fpsyg.2023.1108275

**Published:** 2023-02-06

**Authors:** Carola Dell’Acqua, Daniela Palomba, Elisabetta Patron, Simone Messerotti Benvenuti

**Affiliations:** ^1^Department of General Psychology, University of Padua, Padua, Italy; ^2^Padova Neuroscience Center (PNC), University of Padua, Padua, Italy; ^3^Hospital Psychology Unit, Padua University Hospital, Padua, Italy

**Keywords:** depression vulnerability, psychophysiology, research domain criteria, emotion, risk for psychiatric disorder

## Abstract

Considering that the classical categorical approach to mental disorders does not allow a clear identification of at-risk conditions, the dimensional approach provided by the Research Domain Criteria (RDoC) is useful in the exploration of vulnerability to psychopathology. In the RDoC era, psychophysiological models have an important role in the reconceptualization of mental disorders. Indeed, progress in the study of depression vulnerability has increasingly been informed by psychophysiological models. By adopting an RDoC lens, this narrative review focuses on how psychophysiological models can be used to advance our knowledge of the pathophysiological mechanisms underlying depression vulnerability. Findings from psychophysiological research that explored multiple RDoC domains in populations at-risk for depression are reviewed and discussed. Future directions for the application of psychophysiological research in reaching a more complete understanding of depression vulnerability and, ultimately, improving clinical utility, are presented.

## 1. Introduction

Major depressive disorder (MDD) is a mood disorder that affects psychological and physiological functioning causing an elevated functional impairment and represents a leading cause of disease burden worldwide (World Health Organization, 2017). Symptoms of MDD include depressive mood, anhedonia, appetite changes, sleep disturbances, apathy, psychomotor retardation or agitation, lack of energy, excessive guilt and worthlessness, poor concentration, and suicidal thoughts. According to the Diagnostic and Statistical Manual of Mental Disorders, 5th edition (DSM-5), MDD is defined by the presence of five or more of these symptoms, one of which must be depressed mood or anhedonia causing social and/or occupational impairment. With these criteria, there are 227 possible combinations of symptoms for an MDD diagnosis (Zimmerman et al., 2015). Hence, a few underlying factors may give rise to very different sets of symptoms.

Given the pervasive nature of MDD, improving the early identification of depression risk, and developing strategies to prevent the onset of full-blown depression is a core priority (Wahlbeck and Mäkinen, 2008). For prevention efforts to succeed, it is necessary to identify people at risk early and, ideally, before they become ill. Studying individuals who currently have depression prevents assumptions about whether the observed conditions represent mere correlates of depressive states or reliable markers of its risk. Hence, in the field of clinical psychobiology, researchers are shifting their focus to the study of biomarkers that characterize individuals that have a greater risk to develop a full-blown depressive episode. One reliable risk condition is a parental history of MDD: indeed, adolescents with a parental history of depression are 3–5 times more likely to develop depression themselves (Gottesman and Gould, 2003; Goodman et al., 2011). Other at-risk conditions include individuals with dysphoria, a condition characterized by subclinical depressive symptoms. Last, individuals with past depression but currently free from clinical symptoms represent a risk condition of having a recurrence of the disorder (Michelini et al., 2021). These three conditions (i.e., parental history of MDD, dysphoria, and past depression) are more vulnerable to the development or recurrence of a full-blown depressive episode than the general population, thus representing target conditions to the study of psychobiological markers of MDD (Hardeveld et al., 2010; Laborde-Lahoz et al., 2015). Another way to identify psychobiological markers of a disorder is to conduct longitudinal studies predicting future psychopathology (Raulin and Lilienfeld, 2009). Some researchers have focused on the prevention of MDD by targeting these at-risk conditions with universal psychological treatments and findings have been promising but rather mixed (e.g., Horowitz and Garber, 2006; Brunwasser and Garber, 2016). Efforts to advance effective prevention and treatment strategies might be hindered by our relatively limited understanding of mechanisms implicated in the development and maintenance of depression.

Considering that the “categorical-polythetic” approach provided by the DSM-5 may not allow a clear identification of all at-risk conditions, a viable way to improve our knowledge of the pathophysiological mechanisms linked to depression is to move beyond this approach and, instead, adopt a dimensional approach (Cuthbert and Insel, 2013; Weinberg, 2023). In this context, the National Institute of Mental Health (NIMH) launched the Research Domain Criteria (RDoC) project, which aims at linking biological and physiological mechanisms to clinical phenomena to generate empirically derived, psychobiological markers of psychopathology (Insel et al., 2010; Cuthbert and Insel, 2013). The RDoC assumes that mental disorders are multidimensional disorders observable at different levels of analysis (e.g., from genetics to behavior). The RDoC matrix is rooted in a dimensional approach to mental health and includes six domains: Positive Valence Systems, Negative Valence Systems, Arousal/Regulatory Systems, Cognitive Systems, Sensorimotor Systems, and Systems for Social Processes. The columns of the matrix include the different units of analysis: genes, molecules, cells, circuits, physiology, behavior, and self-report along dimensional neuro-environmental trajectories. The underlying principle is that by integrating different levels of analysis along these dimensions, the RDoC approach will also contribute to the advancement of our understanding of vulnerability to psychopathology (Dillon et al., 2014). Therefore, RDoC dimensions and constructs should not only be considered as a correlate of psychopathology but also of increased vulnerability. To determine whether dysfunctions within RDoC components relate to future psychopathology, conducting studies based on at-risk categories is warranted.

In the “RDoC era,” psychophysiological models have an important role in the reconceptualization of mental disorders and their vulnerability (Shankman and Gorka, 2015). Indeed, psychophysiological studies have highly contributed to the development and refinement of each RDoC dimension for numerous psychopathological conditions. Psychophysiological models cover multiple levels of analysis of constructs of the RDoC (e.g., neural, autonomic, and psychological; Kujawa and Burkhouse, 2017). In addition, psychophysiological measures have several methodological advantages, such as they are non-invasive, well-tolerated, relatively economical biological measures, and can be used in early infancy through old age. In the present narrative review, studies that have employed a wide array of psychophysiological measures for the investigation of RDoC dimensions in at-risk samples will be described. Ultimately, this review emphasizes the relevance that psychophysiology is playing in the refinement of the RDoC matrix in the context of depression risk.

Regarding the methods for studies selection employed in the current narrative review, the focus was on central (event-related potentials, spectral and time-frequency measures, and the startle eyeblink reflex), peripheral (cardiovascular activity and skin conductance) psychophysiological level of analysis as well as other measures related to specific RDoC domains (e.g., pupillometry, cortisol levels, actigraphy). This narrative review includes studies spanning all age groups, but significant attention is posed to studies on childhood and adolescence as they represent vulnerable windows to the development of psychopathology (Jaffee et al., 2002).

## 2. The positive valence systems

The Positive Valence Systems (PVS) are a set of systems involved in anticipating, obtaining, and responding to pleasant or rewarding stimuli (Olino, 2016). Reduced PVS functioning in depression has been evidenced by multiple units of analysis (e.g., self-report, behavioral, and psychophysiological units; McFarland and Klein, 2009; Treadway and Zald, 2011; Treadway et al., 2012; Liu et al., 2014; Hajcak Proudfit, 2015; Nusslock and Alloy, 2017). Indeed, depression is characterized by reduced positive affect, anhedonia, impaired motivational disposition, and reward insensitivity, which could also represent risk factors for the disorder. Indeed, interest has increasingly turned to insensitivity to pleasant or rewarding content as a putative risk factor that precedes depression and may represent a mechanism for the disorder (Kujawa and Burkhouse, 2017; Weinberg, 2023).

### 2.1. Event-related potentials

At the psychophysiological level, deficits in the approach-related brain system can be assessed by measuring neural responses to pleasant or rewarding stimuli through the computation of electroencephalographic (EEG) event-related potentials (ERPs) during specific tasks. For example, the Late Positive Potential (LPP), a positive sustained centroparietal component that begins around 300 ms after stimulus onset, has been found to be particularly important for the study of affective processing. The LPP is often investigated through affective picture viewing paradigms, and it is larger to emotionally arousing (pleasant and unpleasant) relative to neutral stimuli (images of scenes, faces, or words, e.g., Palomba et al., 1997; Cuthbert et al., 2000; Schupp et al., 2007; Dell'Acqua et al., 2022c). Reduced LPP to pleasant stimuli is considered a marker of clinical depression in adults (Grunewald et al., 2019; Klawohn et al., 2021a; for a review see Hajcak Proudfit, 2015) and children (Whalen et al., 2020). Moreover, there is some evidence that the altered LPP may precede the development of the disorder. For instance, reduced LPP amplitude to pleasant pictures has been observed in adults with dysphoria (Benning and Ait Oumeziane, 2017; Moretta et al., 2021), in adults and children with a parental history of depression (Kujawa et al., 2012; Nelson et al., 2015; Moretta and Messerotti Benvenuti, under review^1^), in adults with remitted depression (Allison et al., 2021), and to prospectively predict depression onset (Levinson et al., 2018; Sandre et al., 2019). Specifically, regarding the parental risk for depression, Kujawa et al. (2012) found that even preschool children with a maternal history of depression, but no depressive symptoms, had a reduced LPP to pleasant images relative to controls.

Another ERP component that has been robustly observed to be reduced in depression is reward positivity (RewP, previously referred to as feedback negativity, see Hajcak Proudfit, 2015), a positive feedback-locked frontocentral deflection occurring ~250 ms following the receipt of a reward relative to loss feedback in simple gambling tasks. The RewP represents a valuable and reliable index of reward responsiveness (Hajcak Proudfit, 2015) and there is strong evidence for considering blunted RewP as a vulnerability marker of depression (Kujawa and Burkhouse, 2017). A blunted RewP has been observed in adults and children with a parental history of depression (Foti et al., 2011; Kujawa et al., 2014a,b; Kujawa et al., 2019), siblings with depressive symptoms (Weinberg et al., 2015b), remitted depression (Whitton et al., 2016; Weinberg and Shankman, 2017), and to prospectively predict the first onset of a depressive disorder in adolescent girls with no lifetime depression (Bress et al., 2013; Nelson et al., 2016; Michelini et al., 2021; Burani et al., 2021c). A study that included a large sample of never-depressed adolescent girls reported that reduced RewP amplitude was cross-sectionally related to baseline subclinical depressive symptoms and parental depression history and longitudinally predicted first-onset depressive disorder (Nelson et al., 2016). Moreover, Burani et al. (2021c) showed how a blunted RewP and maternal suicidal thoughts and/or behaviors predicted the onset of depressive symptoms at a 1-year follow-up in a sample of adolescent girls. Notably, within a group of adults with clinical depression, the LPP to pleasant images and RewP predicted a remission status 9 months following the assessment, whereby larger potentials were associated with a higher probability of remission, relative to those that had lower ERPs values (Klawohn et al., 2021b). The reviewed literature suggests that the LPP and the RewP, two distinct measures of neural sensitivity to appetitive cues, might represent early emerging markers of depression vulnerability.

### 2.2. Alpha asymmetry and EEG time-frequency measures

Other studies have examined EEG frequency bands related to approach motivation. A well-established index of affective disposition is frontal asymmetric alpha activity (Davidson, 1998), considered to be inversely related to the level of cortical activation, and an EEG marker of positive affect (Gable et al., 2021). Depression has been associated with an asymmetric pattern of resting-state alpha activity characterized by increased alpha in the left frontal cortex compared to the right, possibly reflecting the hypoactivation of approach-related motivation (Allen et al., 2004; but see also Van Der Vinne et al., 2017). Reduced resting-state left relative to right frontal EEG activity has been observed in unaffected offspring of individuals with MDD (Dawson et al., 1997), and prospectively predicted the onset of depression (Pössel et al., 2008; Nusslock et al., 2011). To date, only a few studies have examined alpha asymmetry during emotional processing in at-risk samples. A study observed reduced left frontal EEG activation (i.e., greater left alpha) to both happy and sad clips in children with a parental history of depression, suggesting that at-risk individuals might have reduced approach motivation during the viewing of all affective cues (Feng et al., 2012; Lopez-Duran et al., 2012). Similarly, individuals with a parental history of MDD showed greater left relative to right frontal EEG alpha activity during a reward-based laboratory task (Nelson et al., 2013). Furthermore, Mennella et al. (2015) found that young adults with, but not without, dysphoria showed reduced alpha desynchronization in the left relative to right anterior sites during an emotional imagery task of pleasant, neutral, and unpleasant narratives, indicating an overall blunted motivation in this at-risk condition. Most of the studies have analyzed alpha activity only at anterior scalp sites, but a smaller alpha desynchronization (i.e., greater alpha) in frontal and right centro-parietal regions to pleasant images was recently found in dysphoria (Messerotti Benvenuti et al., 2019). Given that right parietal activity reflects arousal (Bruder et al., 2005; Stewart et al., 2011), these results were interpreted as an under-engagement of the approach-related motivational system in individuals with dysphoria. Alpha asymmetry during the engagement in emotional tasks remains to be fully explored in depression vulnerability.

Considering the limited and mixed evidence regarding alpha asymmetry in the study of approach motivation in at-risk samples, research has explored other time-frequency correlates of affective processing of pleasant/rewarding stimuli. Delta oscillations are of particular interest as they appear to have a functional role in monitoring the motivational relevance of affective cues and in the identification of pleasant/rewarding stimuli that are generated by subcortical regions involved in the reward system (Knyazev, 2007, 2012; Foti et al., 2015). Blunted delta power to pleasant or rewarding stimuli, which was associated with clinical depression (Foti et al., 2015; Dell’Acqua et al., 2022d), has been observed in individuals with dysphoria (Dell’Acqua et al., 2022a) and to prospectively predict first-onset depressive disorder in a sample of never-depressed adolescent girls at an 18-month follow-up (Nelson et al., 2018). Moreover, Ethridge et al. (2021) explored the intergenerational concordance of delta power to rewards in women with a history of depression and their daughters and found that there was a positive relationship between offspring and mothers’ delta power to rewards. Additionally, they found that having a mother with depression altered the typical increase in reward sensitivity seen during pubertal development, thereby interfering with neural development during this critical period (Ethridge et al., 2021).

### 2.3. Startle eyeblink reflex

Although most psychophysiological contributions to the study of the PVS in depression vulnerability come from EEG studies, other psychophysiological indices have also been useful in exploring this relation. For example, the startle eyeblink reflex consisting of the rapid evoked contraction of the *orbicularis oculi* muscle represents a measure of affective modulation when the startle probe is presented during affective processing mostly 500 ms after the beginning of the presentation of an emotional stimulus. Specifically, the reflex is potentiated during unpleasant affective states and inhibited during pleasant affective states (e.g., Bradley et al., 1999). The absence of startle attenuation to pleasant images, documented in depression (e.g., Dichter and Tomarken, 2008; see Boecker and Pauli, 2019), indicates reduced approach motivation and has also been documented in dysphoria (Mneimne et al., 2008) and in individuals with past but recurrent depression (Vaidyanathan et al., 2014).

### 2.4. Peripheral psychophysiology: Cardiovascular activity and skin conductance

Autonomic nervous system (ANS) changes are measures associated with both affective processing and arousal/metabolic requirements of emotional responding. Cardiac autonomic modulation, as the heart is dually innervated by the two branches of the autonomic nervous systems, can be detected by measuring heart rate that mirrors the sympathetic (acceleration) and parasympathetic (deceleration) nervous systems (Berntson et al., 1993). During active emotional tasks (e.g., imagery, public speaking) vagal withdrawal (i.e., cardiac acceleration) is considered a pattern of autonomic flexibility to respond to stimuli in the environment (Porges, 1997). Cardiac autonomic balance can also, and mostly, be measured through heart rate variability (HRV), a measure of beat-to-beat variation in the heart over time that reflects the balance between the two ANS branches on the heart (Task Force of the European Society of Cardiology and the North American Society for Pacing and Electrophysiology, 1996). Reduced HRV reflects reduced cardiac vagal inhibitory control and has been observed in response to pleasant and unpleasant emotions during active tasks (i.e., imagery, recall of events) in healthy individuals (e.g., Marci et al., 2007; Kreibig, 2010). Individuals with dysphoria showed reduced heart rate increases and less HRV reductions, which reflect inadequate cardiac vagal control, during the imagery of pleasant, but not unpleasant or neutral, scripts relative to controls (Messerotti Benvenuti et al., 2015). In addition, reduced skin conductance response, a measure of the activity of sympathetic cholinergic neurons at the level of eccrine dermal sweat glands (Venables and Christie, 1980), to pleasant stimuli (but also unpleasant) has been shown in individuals with dysphoria relative to a control group (Benning and Ait Oumeziane, 2017; De Zorzi et al., 2021).

### 2.5. Interim conclusion

Taken together, the reviewed psychophysiological findings provide consistent support for a lack of sensitivity to pleasant and rewarding stimuli in vulnerability to depression. More integrative research is needed to clarify which of these measures might be more useful in the early identification of MDD as well as whether they could be leveraged together to improve clinical utility in at-risk samples.

## 3. The negative valence systems

The Negative Valence Systems (NVS) encompass five constructs related to responses to aversive stimuli or events. These constructs include responses to acute threat, potential threat, sustained threat, loss, and frustrative non-reward. Compared to the PVS, data on the reactivity to unpleasant stimuli in depression and vulnerability to depression have been extensively produced in several (and different) research areas and therefore the findings are rather mixed and sometimes even unable to show any significant effect (for a meta-analysis and a review on psychophysiological studies on emotional reactivity see Bylsma et al., 2008 and Bylsma, 2021). Initial theories suggested that depression would be characterized by an increased reactivity to unpleasant emotional stimuli based on the idea that individuals’ background affective state would prime reactivity to a stimulus of matching valence (Rosenberg, 1998; Rottenberg, 2017). Cognitive theories of depression (Beck and Bredemeier, 2016) seem to have a similar hypothesis: negative cognitive schemas guide preferential processing of negative stimuli which, in turn, lead to enhanced attention and intake of these cues. For instance, in support of this claim, individuals with dysphoria, but not controls, repeatedly showed a prolonged cardiac deceleration during passive viewing of unpleasant stimuli as compared with neutral ones, suggesting a sustained intake of unpleasant cues and a mood-related bias in this at-risk group (Messerotti Benvenuti et al., 2020; Moretta et al., 2021). Additionally, children of mothers with MDD showed greater physiological reactivity, indexed by pupil dilation, to sad, but not happy or neutral faces compared to children of non-depressed mothers (Burkhouse et al., 2014). However, the greater processing of unpleasant images observed in dysphoria does not seem to lead to greater action preparation and reactivity. Indeed, from most research using both passive and active tasks and different psychophysiological measures, depression appears to be mostly characterized by a reduced emotional reactivity to unpleasant stimuli (Foti et al., 2010; MacNamara et al., 2016; Hill et al., 2019; for a review see Bylsma, 2021). The lack of reactivity to unpleasant contents is in line with the emotion context insensitivity hypothesis (ECI; Rottenberg et al., 2005; Bylsma et al., 2008; Bylsma, 2021), which suggests that depression might be characterized by an overall blunted emotional reactivity, with reduced psychophysiological responses to all affective cues.

### 3.1. Event-related potentials

In support of the ECI model in depression risk, studies on the LPP during affective picture processing have also observed reduced LPP to unpleasant images in dysphoria relative to a control group (Benning and Ait Oumeziane, 2017; Grunewald et al., 2019), although some studies failed to find this effect (Moretta et al., 2021). Besides, the offspring of parents with a history of MDD had a reduced LPP to unpleasant faces and scenes compared to a control group (Kujawa et al., 2012; Nelson et al., 2015; Moretta and Messerotti Benvenuti, under review^1^). Importantly, in a large longitudinal study (Michelini et al., 2021), blunted LPP to unpleasant stimuli was one of the main predictors of fist-onset depressive disorder over a period of 3 years. However, findings are rather mixed as other studies linked maternal risk for MDD with enhanced LPP to unpleasant images (Speed et al., 2016).

Another way to examine the Negative Valence System is to assess EEG responses to the commission of an error (i.e., error monitoring). Indeed, making a mistake is generally perceived as subjectively unpleasant and, at times, it can be perilous and threatening to one’s life (Weinberg et al., 2016). A specific physiological measure of error monitoring is EEG error-related negativity (ERN), which arises as a negative electrocortical deflection in the ERP at frontocentral scalp sites within 100 ms following the commission of an error vs. a correct response (Gehring et al., 1995). The ERN has been mostly employed in the study of anxiety disorders (e.g., Meyer et al., 2018a), but there is some evidence that the ERN is blunted in adults and children with clinical depression (Ruchsow et al., 2004, 2006; Schrijvers et al., 2008; Weinberg et al., 2015c; Dell'Acqua et al., 2023) and depression risk (Meyer et al., 2018b; Tabachnick et al., 2018). For instance, Meyer and colleagues reported that the offspring of women with recurrent MDD had a reduced ERN relative to a control group, even when accounting for maternal anxiety (Meyer et al., 2018b). Another study showed that subclinical depressive symptoms were linked to blunted ERN in children involved with Child Protective Services (Tabachnick et al., 2018). However, other studies reported greater ERN in clinical depression (Chiu and Deldin, 2007; Holmes and Pizzagalli, 2010). Although these results are promising, more research on multiple at-risk populations is needed to clarify whether a blunted ERN can be considered a psychobiological marker of depression.

### 3.2. Startle eyeblink reflex

Other evidence comes from studies on the startle reflex measured at the *orbicularis oculi* muscle during exposure to emotional cues, which have reported reduced startle potentiation to unpleasant stimuli in individuals with dysphoria (Messerotti Benvenuti et al., 2020) but also enhance startle potentiation in individuals with past but recurrent depression (Vaidyanathan et al., 2014), suggesting that risk may not be equivalent in remitted individuals.

### 3.3. Skin conductance

As noted above in the PVS section, reduced skin conductance during the viewing of all emotional stimuli in individuals with dysphoria relative to a control group was reported (Benning and Ait Oumeziane, 2017). However, while reduced reactivity to unpleasant cues in affective processing tasks may represent a psychobiological marker of depression, greater skin conductance during sad mood induction and recovery were observed in the offspring of mothers with depression relative to a control group (Daches et al., 2020).

### 3.4. Interim conclusion

Taken together, the literature examining the NVS functioning in depression risk is mostly inconsistent and this might be due to several reasons, including cross-study differences in tasks and types of stimuli used, and/or the presence of comorbid anxiety symptoms (for reviews, see Weinberg et al., 2015a; Dickey et al., 2021). Although the role of NVS functioning in vulnerability to depression is not definite, many reviewed psychophysiological studies on emotional reactivity in at-risk samples suggest that vulnerability might be related to blunted responses to unpleasant stimuli, indicating a general pattern of blunted motivation in accordance with the ECI model.

## 4. The arousal and regulatory systems

The DSM-5 criteria for MDD include physical alterations, such as fatigue, sleep disturbances, and appetite changes. Beyond these three bodily symptoms, no other physical symptom is mentioned. However, other somatic symptoms are prevalent in individuals with depression, including headaches, musculoskeletal symptoms, palpitations, and upset stomach (Breslau et al., 2000; Vaccarino A. L. et al., 2008). Arousal might have a primary role in the somatic and neurovegetative symptoms experienced by individuals with depression and they can be ascribable to the Arousal and Regulatory Systems (ARS) of the RDoC (Gunzler et al., 2020). Somatic symptoms of depression are associated with longer disease duration, greater disability, poorer clinical outcomes, and elevated healthcare costs (Vaccarino A. L. et al., 2008; Vaccarino V. et al., 2008). These somatic consequences could partly be due to metabolic, immuno-inflammatory, autonomic, and hypothalamic–pituitary–adrenal axis (HPA) imbalances which can also reflect an altered psychoneurimmumological interaction. These imbalances are often present among MDD patients (Penninx et al., 2013) and they can increase the risk of developing cardiovascular diseases, metabolic syndromes, and overall immune system deterioration (Wolkowitz et al., 2011).

### 4.1. Sleep quality: EEG and actigraphy

Circadian rhythm alterations, such as sleep problems and insomnia, are not only a correlate of MDD but accumulating evidence suggests that they may represent a biomarker of the disorder (Modell and Lauer, 2007; Wiebe et al., 2012). Sleep disturbances are also a typical residual symptom following remission from depression (Carney et al., 2007). For instance, fragmented REM sleep assessed with the EEG (e.g., reduced sleep spindles, shorter latencies to REM, longer REM), was related to subclinical depressive symptoms (Pesonen et al., 2019), was observed in remitted individuals (Jindal et al., 2002), in adolescents and adults with a parental history of MDD (Lopez et al., 2010; Bat-Pitault et al., 2013), and was predictive of depression onset in at-risk adolescents (Rao et al., 2009). Moreover, reduced sleep quality, as assessed by self-report and actigraphy measures, was reported in adolescents with a parental history of MDD (Chen et al., 2012; Wescott et al., 2019), and to prospectively predict depressive symptoms in adolescents (Bei et al., 2015). Notably, altered sleep structure, as assessed with actigraphy and EEG, was observed even in infants born from depressed mothers, suggesting that even the prenatal environment could promote depression vulnerability of the child (Armitage et al., 2009; Bat-Pitault et al., 2017). Other researchers have looked at EEG vigilance and arousal and reported reduced arousal, as indexed by greater posterior resting alpha power in individuals with a parental history of MDD (Bruder et al., 2012).

### 4.2. Peripheral psychophysiology: Cardiovascular activity and skin conductance

Vulnerability to depression has also been related to autonomic unbalances, such as increased heart rate and reduced HRV in resting conditions (in depression: Kemp et al., 2010; Koch et al., 2019; with dysphoria, familial risk, and remitted; Vaccarino V. et al., 2008; Dell’Acqua et al., 2020; Moretta and Messerotti Benvenuti, 2022). Reduced resting HRV in a wide array of at-risk samples suggests that decreased cardiac autonomic balance might serve as an early marker of depression vulnerability. Moreover, a multi-wave study on a large sample of university students showed that a smaller decrease in respiratory sinus arrhythmia (RSA, a measure of vagal activity) and greater increases in heart rate in response to sad clips predicted greater depressive symptoms when individuals encountered negative life events, perhaps due to attenuated self-regulatory abilities (Stange et al., 2017). A reduced cardiac autonomic balance, as indexed by greater parasympathetic activation (reduced decreases in HRV) and sympathetic withdrawal (reduced pre-ejection period), during psychological (e.g., unsolvable puzzle) and physical challenges (e.g., handgrip), was also observed in youths with past depression relative to a control group (Bylsma et al., 2015). Conversely, while individuals with MDD showed blunted RSA and reduced heart rate increase to stress tasks (i.e., cold pressor and speech task), those in remission did not show the same pattern (Salomon et al., 2013; Bylsma et al., 2014), suggesting that the lack of withdrawal of parasympathetic control during stress might be state-dependent and not a putative risk factor of MDD in remitted individuals.

Another psychophysiological measure related to autonomic activity is skin conductance. As previously described, skin conductance mirrors exclusively the sympathetic nervous system activity. Accordingly, during the viewing of pleasant and unpleasant pictures, healthy individuals showed comparable skin conductance responses to similarly arousing stimuli, both pleasant and unpleasant, relative to neutral ones. Instead, individuals with subclinical depression showed reduced skin conductance to all emotional stimuli, supporting both the hypothesis of reduced functioning of the Arousal and Regulatory Systems, as well as the PVS and NVS domains (Benning and Ait Oumeziane, 2017). Similarly, reduced skin conductance response was reported in individuals with depression during a mental arithmetic task (Kim et al., 2019) and in individuals with dysphoria during a public speaking task (Schwerdtfeger and Rosenkaimer, 2011). Additionally, even unaffected offspring of chronically depressed mothers showed reduced skin conductance to stressful situations (i.e., arguments between adults; Cummings et al., 2007).

### 4.3. Cortisol levels

Another measure related to the Arousal and Regulatory domain is cortisol, the main stress hormone that reflects HPA functioning and that has been widely used in the study of neuroendocrine and dysfunctions in MDD (Lopez-Duran et al., 2009; Herbert, 2013). Individuals with depression have been shown to have elevated morning cortisol (e.g., Michael et al., 2000) and a greater cortisol awakening response (CAR; e.g., Bhagwagar et al., 2005; Vreeburg et al., 2009; but see also Huber et al., 2006). Interestingly, increased morning cortisol (e.g., Young et al., 2006; Dougherty et al., 2009) and CAR (e.g., Vreeburg et al., 2010; Nederhof et al., 2015) have been found in never-depressed offspring of parents with a depressive disorder. Moreover, higher CAR cortisol levels were reported in adolescents who subsequently developed a major depressive episode in the following year (Goodyer et al., 2000; Adam et al., 2010; Vrshek-Schallhorn et al., 2013). Collectively, these findings suggest that vulnerability to depression may be related to a hyperactive HPA, mostly in relation to its circadian rhythm (morning cortisol and CAR). The increased activity of the HPA axis is thought to be mostly related to reduced inhibition by endogenous glucocorticoids in the synthesis and release of the adrenocorticotrophic hormone-releasing factor in the paraventricular nucleus and adrenocorticotrophic hormone in the pituitary (Pariante and Lightman, 2008). Regarding cortisol reactivity to a stressor, a relatively blunted cortisol stress reactivity even when controlling for baseline measures was repeatedly observed in MDD (Burke et al., 2005; Harkness et al., 2011). However, whether a blunted cortisol stress reactivity represents a psychobiological marker of depression is rather unclear, but some studies reported reduced cortisol reactivity to stressors in adults and children with dysphoria (de Rooij et al., 2010; Hankin et al., 2010; Suzuki et al., 2013), those with a familial risk for depression (Morris et al., 2017), and in individuals in remission (Morris et al., 2014; but see also Morris et al., 2012; Höhne et al., 2014). However, to our knowledge, cortisol stress reactivity has not been examined in individuals with a parental risk for depression, and whether depression risk relates to blunted cortisol reactivity should be further explored.

### 4.4. Interim conclusion

Collectively, vulnerability to depression seems to be characterized by alterations of the Arousal and Regulatory Systems, such as sleep disturbances and autonomic unbalances in resting and stress-related conditions. As supported by studies on heart rate and cortisol, individuals at-risk for depression seem to be characterized by somatic heightened activation in resting conditions. Studies that assessed cardiovascular reactivity, skin conductance, and cortisol changes to pleasant and unpleasant stimuli (i.e., images, stressors) suggest that at-risk samples might have reduced physiological arousal when mobilization is required. These results emphasize that the Arousal and Regulatory Systems support the affective systems in the PVS and NVS domains and are consistent with the ECI hypothesis of a blunted emotional reactivity (Bylsma et al., 2008; Bylsma, 2021).

## 5. The cognitive systems

In addition to affective and somatic symptoms, cognitive symptoms have been widely reported in individuals with depression. One of the DSM-5 criteria for depression is, indeed, a diminished ability to think, concentrate, or make decisions (American Psychiatric Association, 2013). Cognitive dysfunctions in depression include impairments in cognitive control. Studies have reported that individuals with depressive symptoms show reduced sustained and divided attention (McClintock et al., 2010), overgeneralized declarative memory (Zhou et al., 2017), reduced cognitive flexibility, set-shifting, planning, and updating (Dotson et al., 2020; Dell’Acqua et al., 2022b). These deficits align with the Cognitive Systems domain of the RDoC, which includes constructs of Attention, Perception, Declarative Memory, Language, Cognitive Control, and Working Memory (Insel et al., 2010; Cuthbert and Insel, 2013). Cognitive control deficits have emerged as one of the potential behavioral endophenotypes of depression (Webb et al., 2016). Indeed, cognitive control deficits often persist in remitted individuals (Snyder, 2013), are a stable and reliable characteristic (Sarapas et al., 2012), and showed moderate-to-high heritability (Friedman et al., 2008). In addition, there is some evidence of cognitive control impairments in healthy, unaffected twins at risk for MDD (Christensen et al., 2006).

### 5.1. Event-related potentials

Besides, impairments in the Cognitive Systems are strictly related to the PVS and NVS domains. For instance, numerous studies have investigated cognitive control in affective contexts in relation to depressive symptoms (e.g., Koster et al., 2011; Joormann and Vanderlind, 2014). Although most research on cognitive control typically focuses on behavioral measures, such as reaction times in various tasks (e.g., Go/No-Go, Stroop; Kertz et al., 2019), some studies have explored the electrocortical correlates of cognitive processing in emotional contexts in depression and individuals vulnerable to depression. A task that has been broadly used is the Emotional Go/No-Go. For example, an enhanced No-Go P300 to negative relative to positive faces was positively correlated with depressive symptoms (Zhang et al., 2016) and was observed in individuals with dysphoria but not in controls (Krompinger and Simons, 2009), suggesting that greater processing resources were needed to inhibit the motor response during the presentation of unpleasant stimuli. Indeed, the P300 is an ERP related to attentional processing and resource allocation (Gray et al., 2004). Similarly, in an oddball paradigm, individuals with dysphoria and remitted depression, but not controls, showed greater P300 following sad relative to happy target faces, suggesting that these samples showed more attentional bias to these stimuli (Bistricky et al., 2014). These initial ERPs findings suggest that a mood-related bias may characterize vulnerability to depression when a cognitive effort is required. Contrariwise, Messerotti Benvenuti et al. (2017) showed a reduced Go/No-Go effect for P3 and delta power in response to pleasant and neutral, but not unpleasant, stimuli in individuals with dysphoria relative to non-dysphoric individuals. These findings suggest that individuals with dysphoria need a reduced and/or less effortful response inhibition to pleasant stimuli, supporting the hypothesis of reduced PVS activity. Evidence for reduced attention to pleasant stimuli comes also from eye-tracking studies, which have shown that individuals with past depression and those with dysphoria spent less time attending to pleasant, but not unpleasant images relative to controls (Sears et al., 2010, 2011).

Another electrocortical measure of cognitive control is the ERN, an event-related potential discussed within the NVS domain. Indeed, the ERN does not only reflect sensitivity to an endogenous threat (i.e., commission of an error) but it is also implicated in cognitive control abilities, namely the ability to rapidly detect errors and adaptively regulate actions in a dynamic environment (Weinberg et al., 2016). Some models on the ERN suggest that this measure acts as an early warning signal following the commission of an error evaluating the need to raise cognitive control resources allocated to the task (Weinberg et al., 2016). As previously described, the literature on the ERN in depression and its risk is still conflicting, with studies evidencing reduced (Ruchsow et al., 2004, 2006; Schrijvers et al., 2008; Weinberg et al., 2015a; Meyer et al., 2018b; Tabachnick et al., 2018;Dell'Acqua et al., 2023) or greater ERN (Chiu and Deldin, 2007; Holmes and Pizzagalli, 2010) in these groups.

### 5.2. Interim conclusion

Collectively, from the reviewed studies, the interaction between cognition and the PVS and NVS in determining depression vulnerability becomes evident. Particularly, individuals at-risk for depression seem to have inhibition difficulties of unpleasant stimuli and facilitation of pleasant ones. Taken together, psychophysiological research on cognitive dysfunctions and the interference of emotion on cognitive processing in at-risk populations is still in its infancy and more studies with more heterogeneous paradigms are needed to further identify psychophysiological markers related to the Cognitive Systems of depression vulnerability.

## 6. The sensorimotor systems

Psychomotor disturbances (retardation or agitation) are core features of depression and are included as a diagnostic criterion in the DSM-5. Considering that motor activity (e.g., walking) is needed to increase the chances of rewarding and pleasant events (e.g., meeting some friends or a partner), it is not surprising that psychomotor retardation and reduced gross motor activity are core features of depression (Razavi et al., 2011; Bewernick et al., 2017; Walther et al., 2019; Shankman et al., 2020; Wüthrich et al., 2022). Indeed, motor processes are strictly related to motivational drive and positive emotionality that support approach actions (Walther et al., 2019). These motor disturbances align with the Sensorimotor Systems of the RDoC, a domain that was recently added to the matrix (Garvey and Cuthbert, 2017). The Sensorimotor domain includes four constructs, namely Motor Actions, Agency and Ownership, Habit, and Innate Motor Patterns. Psychomotor retardation can be ascribed to the Motor Actions Construct.

### 6.1. Actigraphy

The assessment of motor disturbances in depression has long been confined to self-report measures and only recently research is shifting toward more objective and ecological measures, such as actigraphy (Walther et al., 2019). Low levels of motor activity, as assessed by a wrist-worn actigraphy, were documented in older adults with remitted depression relative to an age-matched control group (Pye et al., 2021) and were related to subclinical depressive symptoms (Mendlowicz et al., 1999).

### 6.2. EEG spectral features of motor activity

The EEG correlates of motor activity disturbances have only been investigated in clinical depression and have focused on the examination of resting spectral characteristics in relation to psychomotor retardation levels (Nieber and Schlegel, 1992; Cantisani et al., 2015). For example, a left-lateralized pattern of frontal alpha activity was negatively associated with activity levels (assessed with an actigraphy) in individuals with MDD, suggesting that psychomotor retardation may be related to impaired motivational drive (Cantisani et al., 2015). A negative covariance between resting alpha power over motor areas and activity levels was also reported (Nieber and Schlegel, 1992; Cantisani et al., 2015). Considering that alpha power mirrors inhibition of a cortical region, these results might indicate that psychomotor retardation is reflected in reduced motor cortex activity even in conditions of rest, potentially representing a trait feature or these alternations (Cantisani et al., 2015). Overall, it would be valuable to further explore the link between psychomotor retardation and motivation dispositions by means of fine psychophysiological measures (e.g., startle reflex) in depression and its risk.

### 6.3. Interim conclusion

The lack of systematic research on psychomotor disturbances in at-risk samples does not allow for determining whether these disturbances represent a core underlying etiological mechanism of MDD. Studies on risk samples but also longitudinal designs are warranted to better identify whether motor disturbances may represent a viable target for MDD prevention. This would have several advantages, considering that targeting motor functions could be accomplished in different ways (i.e., physical activity, and brain stimulation; Walther et al., 2017).

## 7. The systems for social processes

Depressive symptoms have long been associated with social impairments and poor social functioning (Gotlib and Hammen, 1992). Social impairments are included within the Systems for Social Processes of the RDoC, which include the following domains: Affiliation and Attachment, Social Communication, Perception and Understanding of Self, and Perception and Understanding of Others. Depression is associated with social anhedonia, namely reduced drive for social affiliation, but also with increased sensitivity to social rejection. As might already be evident, this dimension is closely related to the PVS, particularly in the study of depression.

Sensitivity to social rewards has been included in the Affiliation and Attachment domain and can be assessed with the reward positivity component (RewP, see Section 2) during a social feedback task. For example, in the island gateway task, participants play a game in which they are traveling along the Hawaiian Islands and trying to avoid being voted off the island by other (computerized) players whom they are told are age-matched peers (Kujawa et al., 2014a,b). Participants create online profiles, and, in a series of rounds, vote other players on or off the island, while receiving feedback on which players voted them on or off. Participants receive approximately the same number of acceptances and rejections over the course of the task. It has been recently observed that reduced RewP to social rejection during this task significantly predicted the onset of depressive symptoms in a sample of adolescents (Pegg et al., 2019, 2021), suggesting that blunted neural sensitivity to being socially excluded might represent a psychobiological marker of MDD. Similarly, subclinical depressive symptoms were linked to reduced time-frequency delta power to social rewards (Jin et al., 2019). Additionally, other studies indicated a smaller RewP to social acceptance in individuals with depression (Kujawa et al., 2017; Distefano et al., 2018) and those at risk for the disorder (Freeman et al., 2022a,b).

Related to the Cognitive Systems, the offspring of parents with depression recruited more resources to have an optimal performance during a cognitive task (i.e., larger P300) to prevent making a speech in front of an audience (in case of suboptimal performance), thus avoiding social evaluation (Pérez-Edgar et al., 2006). These latter findings are at odds with the hypothesis of reduced sensitivity to social rejection in depression vulnerability. Further research is needed to better parse social functioning and its psychophysiological correlates in depression vulnerability. Further support for reduced sensitivity to social affiliation, individuals with dysphoria showed a reduced increase in heart rate to social rewards relative to a control group (Brinkmann et al., 2014).

## 8. Discussion

**Figure 1:**
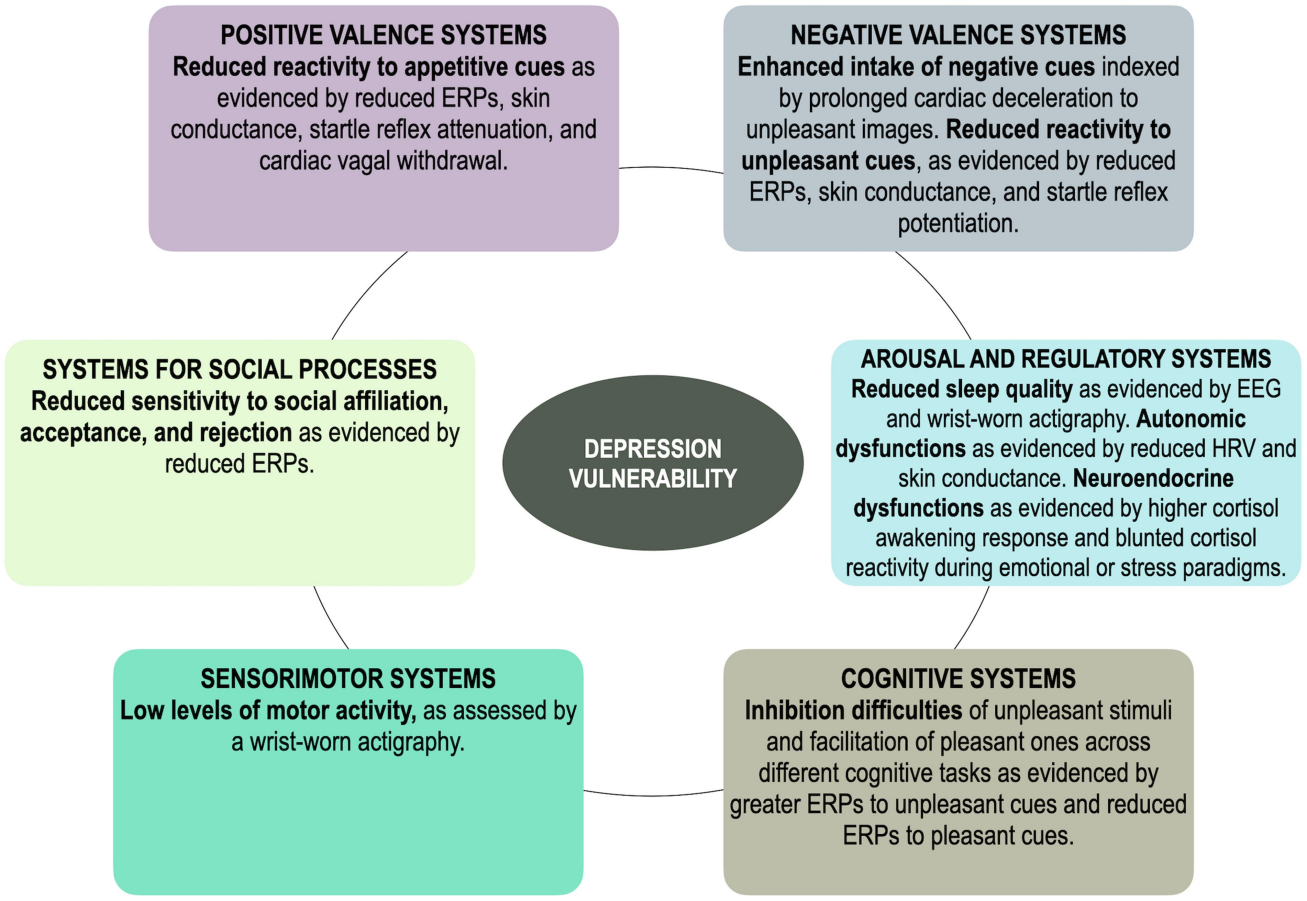
A simplified and schematic illustration of the main findings regarding all RDoC systems in vulnerability to depression. ERPs, event-related potentials.

The present review integrated findings from psychophysiological research in individuals at elevated risk for depression development or maintenance, owing to a familial history, dysphoria, or past depression, as well as longitudinal studies that examined predictors of future depression, adopting an RDoC lens. Hence, each of the described psychophysiological underpinnings could confer a higher risk to develop or maintain full-blown depression and, notably, are apparent before the onset of the disorder (see Table 1; Figure 1 for a summary of the reviewed literature).

**Table 1:** Summary of the studies within each RDoC domain in relation to risk for depression development and maintenance included in the review.

Study	Risk group	Primary findings
*Positive valence systems*		
Benning and Ait Oumeziane (2017) and Moretta et al. (2021)	Dysphoria	Blunted LPP to pleasant stimuli
Moretta and Messerotti Benvenuti (under review)^1^, Nelson et al. (2015), and Kujawa et al. (2012)	Parental history of MDD	
Allison et al. (2021)	Past depression	
Levinson et al. (2018) and Sandre et al. (2019)	Prospective prediction of MDD onset	
Foti et al. (2011), Kujawa et al. (2014a,b), Kujawa et al. (2019), and Weinberg et al. (2015b)	Parental history of MDD	Blunted RewP
Weinberg and Shankman (2017), Whitton et al. (2016)	Past depression	
Burani et al. (2021c), Bress et al. (2013), Michelini et al. (2021), and Nelson et al. (2016)	Prospective prediction of MDD onset	
Dawson et al. (1997)	Parental history of MDD	Reduced resting-state left relative to right frontal EEG activity
Nusslock et al. (2011) and Pössel et al. (2008)	Prospective prediction of MDD onset	
Feng et al. (2012) and Lopez-Duran et al. (2012)	Parental history of MDD	Greater left EEG alpha power to both happy and sad clips vs. neutral
Mennella et al. (2015)	Dysphoria	Reduced frontal left relative to right alpha desynchronization during an emotional imagery task of pleasant, neutral, and unpleasant narratives
Messerotti Benvenuti et al. (2019)	Dysphoria	Smaller alpha desynchronization in frontal and right centro-parietal regions to pleasant images
Dell’Acqua et al. (2022a)	Dysphoria	Blunted time-frequency delta power to pleasant images
Nelson et al. (2018)	Prospective prediction of MDD onset	Blunted time-frequency delta power to rewards
Mneimne et al. (2008)	Dysphoria	Absence of startle attenuation to pleasant images
Vaidyanathan et al. (2014)	Past depression	
Benning and Ait Oumeziane (2017) and De Zorzi et al. (2021)	Dysphoria	Reduced skin conductance response to pleasant (but also unpleasant) images
Messerotti Benvenuti et al. (2015)	Dysphoria	Less heart rate increases and less reductions in HRV during the imagery of pleasant scripts
*Negative valence systems*		
Messerotti Benvenuti et al. (2020) and Moretta et al. (2021)	Dysphoria	Prolonged cardiac deceleration in response to unpleasant stimuli
Burkhouse et al. (2014)	Parental history of MDD	Greater physiological reactivity, indexed by pupil dilation, to sad, but not happy or neutral faces
Benning and Ait Oumeziane (2017) and Grunewald et al. (2019)	Dysphoria	Reduced LPP to unpleasant images
Moretta and Messerotti Benvenuti (under review)^1^, Nelson et al., 2015, and Kujawa et al., 2012	Parental history of MDD	
Moretta et al. (2021)	Dysphoria	No reductions of LPP to unpleasant images
Michelini et al. (2021)	Prospective prediction of MDD onset	Blunted LPP to unpleasant stimuli was one of the main predictors of fist-onset depressive disorder over 3 years
Speed et al. (2016)	Parental history of MDD	Enhanced LPP to unpleasant images
Messerotti Benvenuti et al. (2020)	Dysphoria	Reduced startle potentiation to unpleasant stimuli
Vaidyanathan et al. (2014)	Past depression	Enhanced startle potentiation to unpleasant stimuli
Benning and Ait Oumeziane (2017)	Dysphoria	Reduced skin conductance response to unpleasant stimuli
Daches et al. (2020)	Parental history of MDD	Greater skin conductance during sad mood induction and recovery
Meyer et al. (2018b)	Parental history of MDD	Reduced ERN
Tabachnick et al. (2018)	Dysphoria	
*Arousal and regulatory systems*		
Pesonen et al. (2019)	Subclinical depressive symptoms	Reduced sleep quality (i.e., fragmented REM sleep) assessed with EEG
Jindal et al. (2002)	Past depression	
Bat-Pitault et al. (2013), Bat-Pitault et al. (2017), and Lopez et al. (2010)	Parental history of MDD	
Rao et al. (2009)	Prospective prediction of depressive symptoms	
Armitage et al. (2009), Chen et al. (2012), and Wescott et al. (2019)	Parental history of MDD	Reduced sleep quality assessed with actigraphy
Bei et al. (2015)	Prospective prediction of depressive symptoms	
Bruder et al. (2012)	Parental history of MDD	Reduced posterior cortical arousal indexed by greater posterior resting EEG alpha power
Dell’Acqua et al. (2020)	Dysphoria and past depression	Reduced resting HRV
Moretta and Messerotti Benvenuti (2022)	Parental history of MDD	
Vaccarino V. et al. (2008)	Past depression	
Dougherty et al. (2009), Nederhof et al. (2015), Young et al. (2006), and Vreeburg et al. (2010)	Parental history of MDD	Increased morning cortisol and CAR
Adam et al. (2010), Vrshek-Schallhorn et al. (2013), and Goodyer et al. (2000)	Prospective prediction of MDD onset	Higher CAR cortisol levels predicted MDD onset 1 year later
Salomon et al. (2013)	Past depression	No differences in heart rate reactivity to stressors (speech and cold pressor)
Bylsma et al. (2014)	Past depression	No blunted RSA during a stress
Stange et al. (2017)	Prospective prediction of MDD onset	Smaller decreases in RSA and greater increases in heart rate in response to sad clips predicted greater depressive symptoms when individuals encountered an environmental stressor
Bylsma et al. (2015)	Past depression	Greater parasympathetic activation (reduced decreases in HRV) and sympathetic withdrawal (reduced pre-ejection period), during psychological (e.g., unsolvable puzzle) and physical challenges (e.g., handgrip)
de Rooij et al. (2010) and Suzuki et al. (2013)	Dysphoria	Blunted cortisol levels during stress reactivity paradigms
Morris et al. (2014)	Past depression	
Morris et al. (2017)	Parental history of MDD	
Höhne et al. (2014) and Morris et al. (2012)	Past depression	Greater cortisol levels during stress reactivity paradigms
Schwerdtfeger and Rosenkaimer (2011)	Dysphoria	Reduced skin conductance in a public speaking stress paradigm
Cummings et al. (2007)	Parental history of MDD	Reduced skin conductance to stressful ecological situations
*Cognitive systems*		
Krompinger and Simons (2009) and Zhang et al. (2016)	Dysphoria	Enhanced No-Go P300 to negative faces
Bistricky et al. (2014)	Dysphoria and past depression	Greater P300 following sad targets in oddball task
Messerotti Benvenuti et al. (2017)	Dysphoria	Reduced Go/No-Go effect for P3 and delta power in response to pleasant and neutral, but not unpleasant, stimuli
Meyer et al. (2018b)	Parental history of MDD	Reduced ERN
Tabachnick et al. (2018)	Dysphoria	
*Sensorimotor systems*		
Pye et al. (2021)	Past depression	Low levels of motor activity, as assessed by a wrist-worn actigraphy
Mendlowicz et al. (1999)	Dysphoria	
*Systems for social processes*
Pegg et al. (2019) and Pegg et al. (2021)	Prospective prediction of MDD onset	Reduced RewP to social rejection
Jin et al. (2015)	Dysphoria	Reduced time-frequency delta power to social rewards
Freeman et al. (2022a)	Parental history of MDD	Smaller RewP to social acceptance
Freeman et al. (2022b)	Prospective prediction of depressive symptoms	
Pérez-Edgar et al. (2006)	Parental history of MDD	Recruitment of more resources to have an optimal performance during a cognitive task (i.e., larger P300) to avoid social evaluation
Brinkmann et al. (2014)	Dysphoria	Reduced increase in heart rate to social rewards

CAR, cortisol awakening response; EEG, electroencephalography; ERN, error-related negativity; HRV, heart rate variability; LPP, late positive potential; MDD, major depressive disorder; RewP, reward positivity; RSA, respiratory sinus arrhythmia.

In sum, consistent evidence across multiple psychophysiological levels indicates that reduced responses to appetitive/rewarding cues, belonging within the Positive Valence domain, characterize individuals that are more vulnerable to depression onset. Indeed, based on the reviewed literature, reduced approach motivation seems to represent the most consolidated and robust vulnerability marker of depression. Evidence on the Negative Valence is more mixed, but most studies suggest that at-risk samples may be characterized by a greater intake of unpleasant information that, however, does not lead to greater reactivity but to a blunted reactivity to unpleasant cues. These findings suggest that, in line with the ECI hypothesis (Bylsma et al., 2008), blunted positive and negative emotional reactivity might represent a risk factor for MDD. Depression risk appears to be also characterized by alterations in the Arousal and Regulatory domain, whereby at-risk samples are characterized by sleep alterations and autonomic unbalances in resting conditions and during the viewing of emotional images or stress induction paradigms. Within the Cognitive domain, EEG studies have looked at how emotional cues modulate cognitive resources in depression vulnerability and found that at-risk samples experience inhibition difficulties during the presentation of unpleasant content and facilitated inhibition to pleasant content, suggesting that – at the cognitive level – enhanced attention and processing of mood-related content might be a risk factor of MDD. Moreover, whether motor disturbances that characterize depression, ascribable to the Sensorimotor domain, are viable vulnerability factors of depression still needs to be properly explored, although some studies suggest that low levels of motor activity might characterize at-risk samples. Last, psychophysiological research has recently begun to examine correlates of social processes, but this work is still in its early stages and needs to be extended to other levels of analysis.

From the reviewed literature, what emerges is a strong interrelation among each of the RDoC domains in depression vulnerability. For example, by studying autonomic reactivity (Arousal and Regulatory domain) to unpleasant laboratory stressors (Negative Valence domain), cognitive processes (Cognitive domain) to affective stimuli (Positive and Negative Valence domain), and the relation between psychomotor retardation (Sensorimotor domain) and approach motivation (Positive valence domain) or baseline cortical arousal (i.e., posterior alpha; Arousal and Regulatory domain), researchers are concurrently tackling several dimensions related to depression vulnerability. Indeed, it becomes clear that vulnerability may not be conferred by a single process but by the interrelation of many processes. This highlights how the development of a single condition is truly a product of the interplay among multiple factors that can be potentially targeted for prevention and early intervention.

Nevertheless, another important aspect that, according to the RDoC framework, has a transversal impact on all domains is environmental influences. For example, exposure to negative stressful life events is a well-established risk factor for psychopathology and seems to have an impact on multiple domains. Chronic stress has significant adverse effects on brain regions implicated in reward processing (Pizzagalli, 2014; Ethridge et al., 2018; Burani et al., 2021b) and endocrine and autonomic regulation (Sheth et al., 2017), processes that have been described throughout this review. Of note, there is evidence of how stressful life events interact with neural activity to rewards to prospectively predict the development of depression (Burani et al., 2021a), further supporting the role of an environmental influence on the functioning of an RDoC domain in determining vulnerability for psychopathology. Although this was not the focus of this review, many other environmental factors may act as catalysts for vulnerability factors in determining the development of depression (Bronfenbrenner and Morris, 2007).

Future work should aim at incorporating multiple dimensions to identify narrower and specific vulnerability profiles to ultimately improve the ability of clinicians to recognize people early and implement *ad-hoc* strategies (e.g., Craske et al., 2016). However, to do this, some issues in the pursuit of psychophysiological vulnerabilities of depression will have to be addressed. Firstly, for the assessment of each RDoC domain, it is important to unify paradigms and methods to promote the replicability of results and build robust evidence across investigations. Then, it is also important to account for sociodemographic variables that may drive some of the mixed findings, such as gender, race, and socioeconomic status. Further, to precisely identify vulnerability profiles, more longitudinal investigations examining trajectories of risk are warranted. Another important point that should be further expanded is the role of development, emphasized in the RDoC model. In particular, the RDoC framework advises posing attention to the importance of improving the knowledge of typical and atypical developmental trajectories as well as enhancing prevention and intervention efforts by identifying reliable and valid biomarkers of risk for psychopathology in early in life. The current narrative review focused on the available studies that included a broad age range, including studies on children, but future efforts should be made to conduct more research on depression risk during early life. Additionally, the present review focused on studies that employed psychophysiological models as these represent a useful framework in redefining dimensions involved in psychopathology and present numerous methodological advantages (e.g., non-invasive, well-tolerated, and economic). However, there are still some barriers to these methods in improving the understanding of psychopathology among minoritized races and ethnicities (e.g., Kredlow et al., 2017; Choy et al., 2022).

In conclusion, the present work described, for each RDoC domain, studies aimed at identifying psychobiological markers of depression risk. Insights into some viable mechanisms that contribute to the development of depression in at-risk samples were provided and the effectiveness and potential of psychophysiological models within the RDoC framework for exploring and understanding depression pathophysiology were emphasized. Nonetheless, despite the significant progress that has been made, additional effort is required to better identify vulnerability profiles that can precisely predict the disorder.

## Author contributions

CDA, EP, DP, and SMB: conceptualization. CDA: writing the original draft, editing, and reviewing. EP, DP, and SMB: supervision, review, and editing. DP and SMB: funding acquisition. All authors contributed to the article and approved the submitted version.

## Funding

This study was supported by a grant from MIUR [Dipartimenti di Eccellenza DM 11/05/2017 n. 262] to the Department of General Psychology, University of Padua, and by a grant from MIUR [PRIN n. 2017BC4MST] to DP. SMB’s work was supported by the University of Padua under the 2019 STARS Grants programme [Acronym and title of the project: A-CAOS-BIRD – Asymmetries and Connectivity in Alpha OScillations: toward Biomarkers of Intergenerational Risk for Depression].

## Conflict of interest

The authors declare that the research was conducted in the absence of any commercial or financial relationships that could be construed as a potential conflict of interest.

## Publisher’s note

All claims expressed in this article are solely those of the authors and do not necessarily represent those of their affiliated organizations, or those of the publisher, the editors and the reviewers. Any product that may be evaluated in this article, or claim that may be made by its manufacturer, is not guaranteed or endorsed by the publisher.

## Footnotes

^1^Moretta, T., and Messerotti Benvenuti, S. (under review). Emotional processing in individuals with familial risk for depression: an ERP and cardiac deceleration study.
